# Cardiac Surgery in the Time of Coronavirus

**DOI:** 10.21470/1678-9741-2020-0161

**Published:** 2020

**Authors:** Mauro Del Giglio, Gabriele Tamagnini

**Affiliations:** 1 Department of Cardiac Surgery, Villa Torri Hospital Bologna, Emilia-Romagna, Italy.

Worldwide we are facing the novel coronavirus outbreak; in many countries, the health systems are under a tremendous pressure due to the lack of preparation. Speaking about cardiac surgery units, the pandemic affects our daily routine in different ways: limited intensive care unit (ICU) beds and ventilation sites, necessity to postpone elective and/or complex cardiac surgeries, shortage of healthcare workers, sick healthcare staff and/or risk of infection of our Teams, risk of developing COVID-19 after cardiac surgery, and patients with COVID-19 needing urgent cardiac operations without having a properly organized operating room and ICU.

The first data available in the literature highlight two issues that link coronavirus infection and cardiovascular disease: patients with COVID-19 have cardiovascular comorbidities in 15% of cases^[1,2]^ and the presence of heart disease appears to play as a risk factor for developing more severe COVID-19 cases^[3]^. On the other hand, some in-hospital patients might face the risk of infection by SARS-CoV-2, with a possible fatal outcome during the perioperative period^[4]^: based on the central role of interleukin-6 and other pro-inflammatory mediators to cause tissue damage during the more severe COVID-19 cases, it’s reasonable that during the postoperative period - normally characterized by a pro-inflammatory state - the patient who gets infected by SARS-CoV-2 would face an increased risk of severe COVID-19^[5]^.

However, Onder et al.^[6]^ reported a case-fatality rate updated to March 17^th^, 2020, that depicts the current situation in Italy: the data analyzed by age group showed a rate of death of 12.8% in 70-79-year-old patients and 20.2% in > 80-year-old patients. It is interesting that those age groups are the ones more often involved in cardiac surgery procedures.

So, clinically and ethically we have to decide which patient can be delayed, but are we sure of the COVID-19 emergency duration? Which pathology can wait? Is it better to treat a young and relatively healthy patient rather than elderly patients to shorten the ICU length of stay (LOS) and all the resources?

As Cardiac Surgeons, we treat every day potentially life-threatening conditions: we sought directions in the National Societies of Cardiac Surgery and, although there are evidences linking the COVID-19 with a myocardial injury and how a patient with acute myocardial infarction should be correctly addressed^[7,8]^, nothing has been clearly said about the triage process in severe valvular diseases.

Matt and Maisano stated in a recent article on PCRonline.com: *“We think that patients with acute coronary syndrome in case of severe coronary artery disease (e.g. severe left main trunk stenosis, severe triple vessel disease with high SYNTAX score) who are not eligible for conservative or interventional treatment may be operated on. This may be true also for younger patients with symptomatic severe aortic valve stenosis, left-sided endocarditis with a severe valve defect and/or large mobile vegetation, large ascending aortic aneurysm (>6 cm in diameter), and symptomatic severe mitral valve insufficiency.”.*

We agree with that position but what about “elective surgery”? Should we rethink that definition?

Going through the recommendations of the United Kingdom’s National Health Service and Society for Cardiothoracic Surgery in Great Britain and Ireland^[9]^ and the American College of Surgeons^[10]^, there is no mention to which kind of patients should be preferably operate on, but definitely there are some advices to optimize the treatment, such as shorten as much as possible the LOS, minimize the blood loss^[11]^, minimize the risk of exposure to SARS-CoV-2 for patients and staff, and pay attention to in-patient bed capacity (postponing elective cases which require in-patient resources will preserve those resources for acute needs).

Our Team has decided to centralize the patients in the GVM Cardiovascular Hub Center, the Maria Cecilia Hospital in Cotignola, Ravenna, Italy. We’re screening every patient with swab tests for potentially positivity to SARS-CoV-2: our priority is to assure a coronavirus-free environment.

At the moment, we treat in our Center:

- patients with coronary artery diseases and severe symptoms (low-threshold angina) or bad localization of the disease (who are not eligible for interventional procedures);
- patients with severe valvular diseases and symptoms and/or signs of cardiac function impairment;
- generally every patient who can’t clinically have his/her procedure delayed for more than two or three months.

## CONCLUSION

The novel coronavirus outbreak is putting a lot of pressure in our health systems; as Surgeons, we are called for optimizing our activity in order to spare resources and to stop spreading the infection. On the other hand, the population who is at risk of severe COVID-19 cases has about 15% of cardiovascular comorbidities and about the same age profile of the cardiac surgery population. We think it’s mandatory to consider epidemiologic studies to conceive a new concept of elective cardiac surgery, in order to safely treat patients before they get infected.

What do you do in your Center? Any experience and knowledge to optimize patient triage?
